# Effects of High-Fat Diet on Stress Response in Male and Female Wildtype and Prolactin Knockout Mice

**DOI:** 10.1371/journal.pone.0166416

**Published:** 2016-11-28

**Authors:** Manu Kalyani, Kathryn Hasselfeld, James M. Janik, Phyllis Callahan, Haifei Shi

**Affiliations:** 1 Department of Biology, Physiology and Neuroscience, Miami University, Oxford, Ohio, United States of America; 2 Cell, Molecular, and Structural Biology, Miami University, Oxford, Ohio, United States of America; Brock University, CANADA

## Abstract

Prolactin (PRL) is well characterized for its roles in initiation and maintenance of lactation, and it also suppresses stress-induced responses. Feeding a high-fat diet (HFD) disrupts activity of the hypothalamic-pituitary-adrenal (HPA) axis. Whether PRL regulates HPA axis activation under HFD feeding is not clear. Male and female wildtype (WT) and PRL knockout (KO) mice were fed either a standard low-fat diet (LFD) or HFD for 12 weeks. Circulating corticosterone (CORT) levels were measured before, during, and after mice were subjected to an acute restraint stress or remained in their home cages as no stress controls. HFD feeding increased leptin levels, but the increase was lower in KO than in WT mice. All stressed female groups and only LFD-fed stressed males had elevated CORT levels compared to their no stress same-sex counterparts regardless of genotype. These results indicated that HFD consumption blunted the HPA axis response to acute stress in males but not females. Additionally, basal hypothalamic CRH content was lower in HFD than LFD males, but was similar among female groups. Furthermore, although basal CORT levels were similar among KO and WT groups, CORT levels were higher in KO mice than their WT counterparts during stress, suggesting that loss of PRL led to greater HPA axis activation. Basal PRL receptor mRNA levels in the choroid plexus were higher in HFD than LFD same-sex counterparts, suggesting activation of central PRL’s action by HFD feeding in both males and females. Current results confirmed PRL’s roles in suppression of the stress-induced HPA axis activation. Although HFD feeding activated central PRL’s action in both sexes, only the male HPA axis was dampened by HFD feeding.

## Introduction

Prolactin (PRL), synthesized in and secreted from anterior pituitary lactotrophs, is a 23-kDa polypeptide hormone that regulates multiple reproductive and metabolic functions [1,2]. Stress produces well-characterized neuroendocrine responses including activation of the hypothalamic-pituitary-adrenal (HPA) axis. Specifically, stress increases hypothalamic corticotrophin releasing hormone (CRH) neuronal activity in the paraventricular nucleus (PVN), which in turn stimulates the anterior pituitary to produce adrenocorticotrophic hormone (ACTH) and ACTH stimulates the adrenal cortex to secrete corticosterone (CORT) into the circulation in rodents [3]. In addition to HPA axis activation, stress also increases PRL levels in the plasma [4–6] and within the hypothalamus [6]. Circulating PRL enters the brain via a carrier-mediated transport mechanism by binding to the long splice form of the PRL receptor (PRLR) located in the choroid plexus [7–9]. Within the brain, PRL dampens stress-induced activation of the HPA axis and exerts protective effects against stress, as lateral cerebroventricle administration of PRL decreases stress-induced ACTH secretion [6,10,11] and protects against stress-induced hypoglycemia and ulcerogenesis [12]. In contrast, down-regulation of PRLR in the choroid plexus by antisense oligonucleotide treatment elevates ACTH secretion and anxiety-like behavior [6,11]. These studies collectively indicate that PRL suppresses stress-induced HPA activation in the CNS.

There is a complex link between consumption of a high-fat diet (HFD) and alterations in HPA axis activation. Consuming comfort food, such as high-fat and/or high-sugar diets, reduces HPA axis activity in male rats [13]. The effects of HFD on the activity of HPA axis have been evaluated in mouse models, but their results are conflicting due to differences in methodology and experimental conditions, such as various mouse strains, different housing and sampling conditions, dissimilar HFD contents and feeding durations [14]. In some studies HFD feeding is considered as a stressor since it increases resting circulating CORT levels and enhances HPA responses to stress in rodents [15–17]; whereas in other studies HFD feeding down-regulates activity of the HPA axis by decreasing CRH mRNA in the PVN at the beginning of light phase [18] and reducing resting CORT concentrations at the beginning [19], middle [18], or end [20] of light phase of non-stressed mice. Thus, HFD consumption induces complex changes in the diurnal regulation of different components of the HPA axis.

The mechanisms by which HFD consumption disrupts HPA axis are not fully understood. HFD feeding may change PRL’s effects, which may disrupt HPA axis activity. The interaction between HFD consumption and PRL in stress-induced HPA activation was explored in the current study. We hypothesized that PRL and HFD consumption affected HPA axis activation in response to an acute restraint stress. There are sex differences in the regulation of PRL secretion, with females having higher PRL circulating levels [21] and higher PRL pituitary content [22] than males. Additionally, the function and activation of HPA axis differ between males and females [23]. Therefore, both male and female PRL knockout (KO) mice and their wild-type (WT) littermates fed with a standard low-fat diet (LFD) or a HFD were used to test this hypothesis. Furthermore, previous studies have reported impact of PRL on circulating leptin levels [24], CRH content in the hypothalamus [25], and PRLR expression in the choroid plexus [8,11]. In the current study, these levels were measured in male and female WT and PRL KO mice fed with different diets to indicate roles of PRL deficiency and/or HFD feeding as well as potential sex differences in these measurements.

## Materials and Methods

### Animals and diets

A breeding colony of littermates on 129/Sv background [26] (breeder pairs and RT-PCR sequences provided by Dr. Nelson Horseman, University of Cincinnati) was maintained in a clean, stress-free environment under conditions of controlled lighting (12 h:12 h light:dark cycle, lights on at 0600) and temperature (22–24°C) and had *ad libitum* access to water and a standard rodent LFD. It is noteworthy that PRL deficiency leads to pituitary hyperplasia and undetectable PRL bioactivity in pituitaries; whereas other pituitary hormones are normal in PRL KO mice [26]. PRL deficiency does not affect normal growth at any age or cause any other organ pathology or lesion at gross and macroscopic levels [26]. We confirmed that PRL levels were not significantly different between WT and heterozygous mice of the same sex, with lactating WT and heterozygous females having similarly high levels; PRL was undetectable in male and female PRL KO mice; and pituitary ACTH level was not significantly different between genotypes for either sex. PRL KO females are infertile. Mice were bred by mating heterozygous females with KO males. WT and heterozygous females lactated but KO females did not, indicating that WT and heterozygous females had sufficient PRL levels for lactation. Consequently heterozygous females were used for breeding without using surrogate mothers.

Mice at 3–4 weeks of age were genotyped with RT-PCR using the DNA extracted from ear-punched tissue. PRL forward primer is 5’-ATGGTGGATTAGCCGGAAGT-3’, PRL reverse primer is 5’-TTTCCATGAGTCGGAAAAGC-3’, and neomycin cassette in the transgenic mice is 5’-ATTGCATCGCATTGTCTGAG-3’ [26]. All reactions were incubated at 95°C for 15 min, followed by 35 cycles of 95°C for 20 sec, 35 cycles of 72°C for 60 sec, and 1 cycle of 72°C for 5 min [27]. PCR products were separated on agarose gel by gel electrophoresis, with a single product at 200 bp indicating WT, a single product at ~400 bp indicating KO, and both products indicating heterozygote. All mice were housed individually after genotyping.

At 6 weeks of age, about half of the mice of each genotype were remained on the LFD (3.02 kcal/g; 13.5% fat, 28.5% protein, 58% carbohydrates; LabDiet 5001, St. Louis, MO) and the rest were fed with a HFD (4.73 kcal/g, 45% fat, 20% protein, 35% carbohydrates; Research Diets D12451, New Brunswick, NJ) for another 12 weeks. The LFD and HFD had closely matched amounts of proteins (LFD: 0.239 g/g, HFD: 0.237 g/g) and carbohydrates (LFD: 0.404 g/g, HFD: 0.414 g/g), but different amounts of fat (LFD: 0.05 g/g, HFD: 0.236 g/g). There were four groups for each sex, LFD-fed WT (WT LFD), HFD-fed WT (WT HFD), LFD-fed KO (KO LFD) and HFD-fed KO (KO HFD), with 25–30 mice in each group. At the end of 12 weeks of feeding, mice of each diet group were assigned to stress (S) or no stress control (NS) groups with matched body weights and body compositions, with 12–17 mice in each group. All procedures were approved by the Institutional Animal Care and Use Committee at Miami University Ohio.

### Determining stages of estrous cycle

The progression of female estrous cycle was monitored by studying vaginal cytology in WT and KO mice. Although female PRL KO mice underwent cycles that displayed all of the phases, the patterns were irregular. Vaginal smears were performed until all females completed the estrous cycles, starting and ending at the same phase with at least one of each phase. The vaginal smears containing cells were obtained between 1000 and 1200 hours, and stained with DipQuick Stain kit (Jorgensen Laboratories, Inc., Loveland, CO). The stage of the estrous cycle was then determined. Estrus was characterized by large clumps of non-nucleated cornified cells; metestrus was characterized by leukocytes mixed with other cell types; diestrus was characterized by leukocytes without nuclei; and proestrus was characterized primarily by nucleated larger round cells. To reduce variation of HPA activation caused by steroid hormones, all females were studied in diestrus when estrogen levels were relatively low.

### Body weight, caloric intake, and body composition

Body weight was monitored weekly and caloric intake was monitored biweekly. Body composition, including fat mass and lean mass, was assessed biweekly using an EchoMRI body composition analyzer (EchoMedical Systems, Houston, TX) during the 12-week feeding period.

### CORT levels during acute restraint stress or control condition

Stress (S) and no stress (NS) mice were tested in the same procedure room with same housing condition. Restraint stress was performed between 0730 and 0930 to avoid any circadian variation in CORT levels. Serial blood samples were taken via tail clip using microvette tubes (Sarstedt, Newton, NC) before (0 min), at 5 min, 15 min, and 30 min while the mice were restrained (S) or remained in their home cages (NS). Each blood sample was taken in less than one minute. The stressed mice were returned to their home cages immediately after 30 min of stress. All mice were sacrificed 30 minutes later by rapid decapitation and blood samples were taken by decapitation at 60 min for the measurement of CORT levels. Plasma was collected following centrifugation of blood samples and stored at -20°C until they were measured for CORT. CORT levels were quantified using double antibody radioimmunoassay (MP Biomedicals, Santa Ana, CA), with inter-assay variability <8% and intra-assay variability <3%.

### Leptin

The relationship between PRL and leptin, a hormone synthesized in adipose tissues and involved in the regulation of energy balance, is unclear. Previous studies have reported that leptin levels are higher in PRL overexpressing females [24] and PRL KO males [28] compared with their WT littermates, whereas leptin levels are similar between female PRL KO and WT mice [28]. Other studies using PRLR-deficient mouse models have reported either lower leptin levels [29] or no difference in leptin levels [30] compared with their WT counterparts. To corroborate leptin levels between WT and PRL KO mice independent of stress, plasma leptin levels were quantified from all NS groups using a mouse specific enzyme-linked immunosorbent assay (Crystal Chem Inc., Downers Grove, IL), with intra- and inter-assay variability < 10%.

### Hypothalamic CRH levels

The brains of NS groups were removed and hypothalami were collected for CRH extraction. Briefly, the hypothalami were heated at 72°C for 10 min in 1ml of 0.1M acetic acid, cooled on ice, and homogenized. The homogenates were centrifuged at 13,000 g for 15 min at 4°C. An aliquot of 50μl supernatant from each sample was measured for protein concentration using a BCA kit (Thermo Scientific, Rockford, IL) and the remaining supernatant from the sample was lyophilized, reconstituted using the buffer provided by the CRH radioimmunoassay kit (Phoenix Pharmaceuticals, Burlingame, CA), and measured for CRH concentration with inter-assay variability <17% and intra-assay variability <12%. Hypothalamic CRH levels were presented as CRH concentration/protein concentration.

### PRLR expression in the choroid plexus

To measure PRLR gene expression in the choroid plexus of the brains of the NS groups, the lateral ventricles were exposed, and bilateral choroid plexus tissues from 4 mice of the same groups were pooled to ensure sufficient levels of mRNA for quantitative PCR. Total RNA was isolated using Maxwell 16 LEV simplyRNA tissue kit (Promega, Madison, WI). Following turbo DNAse treatment (Ambion, Austin, TX), cDNA was synthesized using 0.5 μg of RNA and Improm-II^™^ reverse transcription system (Promega) and purified using Qiaquick nucleotide removal kit (Qiagen, Valencia, CA). The mouse long form PRLR forward primer is 5’-AAGCCAGACCATGGA TACTGGAG-3’ and the reverse primer is 5’-AGCAGTTCTTCAGACTTGCCCTT-3’ [31]. Housekeeping gene mouse ribosomal protein L7 was used as an endogenous control to indicate relative quantification of mRNA from every sample. The L7 forward primer is 5’-GAAGCTCATCTATGAGAAGGC-3’ and reverse primer is 5′-AAGACGAAGGAGCTGCAGAAC-3’. Quantitative RT-PCR was conducted in triplicates using a Rotorgene 3000 (Corbett Life Science, Sydney, Australia) and Quantitect SYBR mix (Qiagen), with 2-step amplification (95°C for 5 sec) and annealing (60°C for 10 s and 72°C for 15 s) for 48 cycles. The amplified products were confirmed via gel electrophoresis and melt curve analysis. Values obtained for PRLR gene was normalized to L7.

### Statistical analysis

Data were reported as mean ± SEM. All analyses were performed using SAS version 9.2 for Windows. Body weight, body composition, and caloric intake were analyzed using hierarchical analysis of covariance models. Levels of CORT, leptin, CRH, and PRLR were analyzed using a mixed-design analysis of variance (ANOVA) and pre-planned contrasts for dietary effects between genotypes and genotype effects between diets were constructed and analyzed. All tests were performed at the 0.05 level of significance, and Bonferroni adjustments were applied to control for type 1error inflation across multiple comparisons.

## Results

### Body weight and caloric intake

**Fig 1:**
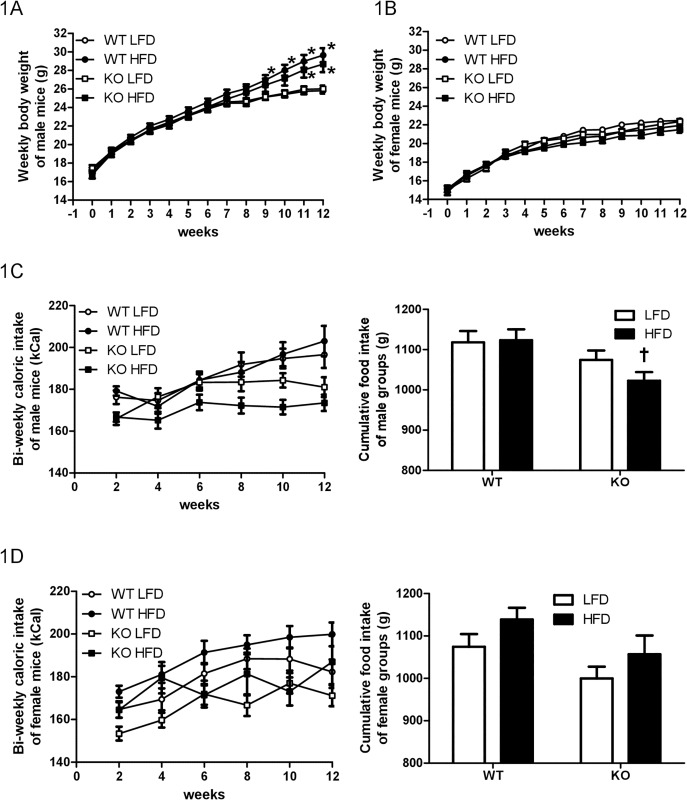
Body weight and caloric intake. Body weight of male (A) and female (B) and caloric intake of male (C) and female (D) wildtype (WT) or prolactin knockout (KO) mice fed with a low-fat diet (LFD) or a high-fat diet (HFD). * Different from LFD-fed males with the same genotype at the same time points. Male: WT chow n = 28; WT HFD n = 26; KO chow n = 32; KO HFD n = 30. Female: WT chow n = 25; WT HFD n = 29; KO chow n = 28; KO HFD n = 26.

Mice of all groups had similar body weights at the beginning of feeding (week 0). WT HFD males gained more weight than WT LFD males between weeks 9 and 12, and KO HFD males gained more weight than KO LFD males during weeks 11 and 12 (Fig 1A). There were main effects of time [F(12,1430) = 217.11, *P* < 0.0001] and group [F(3,1430) = 22.88, *P* < 0.0001], and there was a significant group by time interaction for body weights of male mice [F(36,1430) = 2.43, *P* < 0.0001]. In contrast, LFD and HFD groups of WT or KO female mice did not differ on the measure of their body weights throughout 12 weeks of feeding (Fig 1B). Although there was a main effect of time [F(12, 1482) = 250.34, *P* < 0.0001], there was no significant interaction yielded for female body weights [F(36, 1482) = 0.97, *P* = 0.51]. Consuming a HFD had no effect on caloric intake during any two-week period within the same genotype in males (Fig 1C) or females (Fig 1D). There was no interaction between time and group factors for biweekly caloric intake in males [F(15, 660) = 1.33, *P* = 0.18] or females [F(15, 684) = 0.80, *P* = 0.68]. Although there was no interaction between genotype and diet for 12-week cumulative caloric intake in males [F(1, 110) = 1.28, *P* = 0.26] or females [F(1, 114) = 0.01, *P* = 0.91], *post hoc* tests revealed main effects of genotype on cumulative caloric intake in both males [F(1, 110) = 8.30, *P* < 0.01] and females [F(1, 114) = 5.77, *P* < 0.05]. Specifically, 12-week HFD intake was lower in KO males than WT males [t = 2.84, *P* < 0.05] (Fig 1C), but was similar between WT and KO females [t = 1.77, *P* > 0.05] (Fig 1D). Cumulative LFD intake was similar between WT and KO males [t = 1.24, *P* > 0.05] and females [t = 1.63, *P* > 0.05].

### Body composition

**Fig 2:**
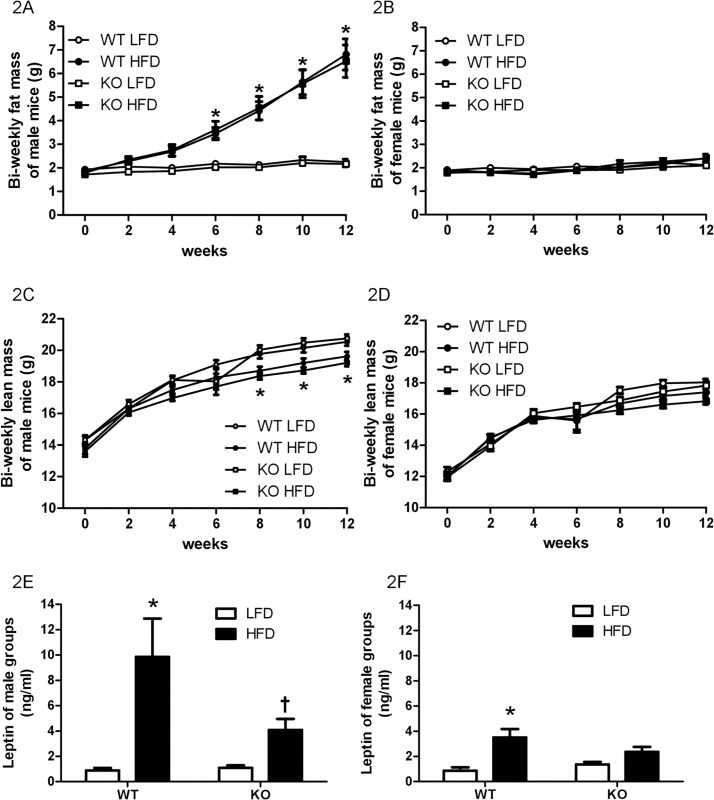
Body composition and plasma leptin levels. Body fat mass of male (A) and female (B), lean mass of male (C) and female (D), and circulating leptin levels of male (E) and female (F) wildtype (WT) or prolactin knockout (KO) mice fed with a low-fat diet (LFD) or a high-fat diet (HFD). A: * Different from LFD-fed groups with the same genotype. C: * Different between LFD-fed KO males and HFD-fed KO males at the same time points. E, F: * Different from LFD-fed groups with the same sex and same genotype. † Different from levels in WT groups with the same sex and diet.

Fat mass was similar at the beginning of feeding (Fig 2A). Regardless of genotype, HFD-fed males had greater adiposity than LFD-fed males after 6 weeks of HFD feeding, whereas HFD- and LFD-fed females had similar fat mass, suggesting that there was no genotype difference in the effect of HFD consumption on fat mass. There was a significant interaction between time and group factors for fat mass in males [F(18, 770) = 10.59, *P* < 0.0001]. In contrast, lean mass was less in KO HFD males compared to KO LFD males but was similar between WT LFD and WT HFD males (Fig 2C). There were main effects of time [F(6, 770) = 195.25, *P* < 0.0001] and group [F(3, 770) = 22.15, *P* < 0.0001] yielded by the ANOVA, but there was no significant interaction between time and group factors [F(18, 770) = 1.14, *P* = 0.31], for lean mass in males. Fat mass and lean mass were similar between LFD- and HFD-fed female groups within the same genotype (Fig 2B and 2D). The ANOVAs yielded no significant interactions for female fat mass [F(18, 798) = 1.29, *P* = 0.19] and lean mass [F(18, 798) = 1.38, *P* = 0.14]. Thus, regardless of genotype, HFD-fed males had more fat mass than LFD-fed males whereas HFD- and LFD-fed females had similar fat mass, suggesting that there was no genotype difference in the effect of HFD consumption on body composition.

### Leptin

**Fig 3:**
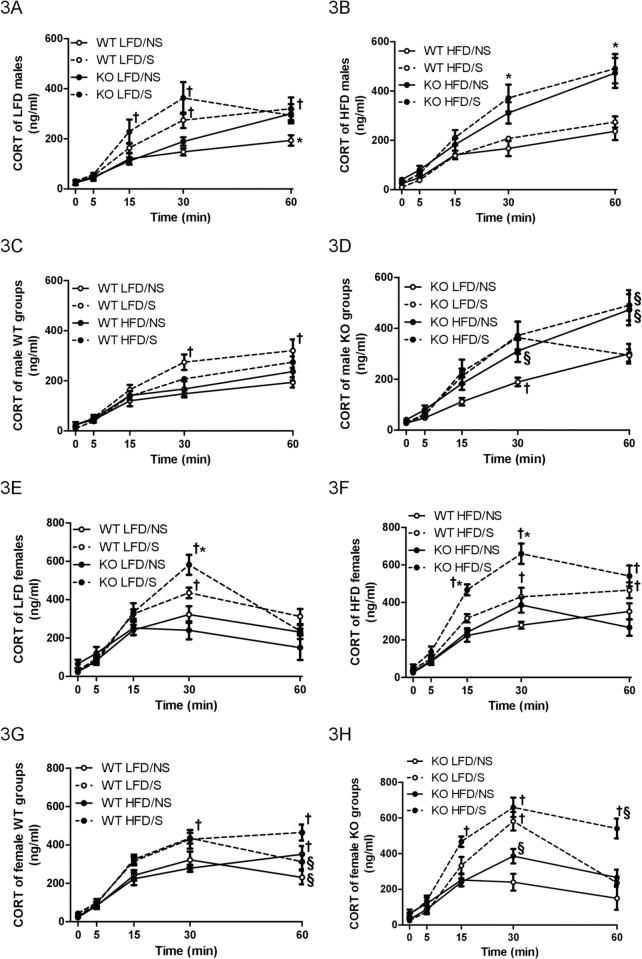
Plasma corticosterone levels. Circulating corticosterone (CORT) levels of male wildtype (WT) and prolactin knockout (KO) mice fed with a low-fat diet (LFD) (A) or a high-fat diet (HFD) (B), and WT and KO males fed with a LFD (C) or a HFD (D), during 30 min restraint stress (S) or no stress control (NS). Circulating CORT levels of female WT and KO mice fed with a LFD (E) or a HFD (F), and WT and KO females fed with a LFD (G) or a HFD (H), during 30 min restraint stress (S) or no stress control (NS). A, B, E, F: * Different between genotypes with the same stress condition. † Different between stress conditions with the same genotype. C, D, G, H: § Different between diets with the same stress condition. † Different between stress conditions with the same diet. Male: WT LFD/NS n = 13; LFD/S n = 15; HFD/NS n = 13; HFD/S n = 13. KO LFD/NS n = 17; LFD/S n = 15; HFD/NS n = 14; HFD/S n = 16. Female: WT LFD/NS n = 12; LFD/S n = 13; HFD/NS n = 14; HFD/S n = 15. KO LFD/NS n = 13; LFD/S n = 15; HFD/NS n = 12; HFD/S n = 14.

Leptin levels were similar between LFD-fed WT and KO same sex groups (Fig 3), consistent with their similar body fat mass. Plasma leptin levels were higher in WT HFD than WT LFD males [t = 3.80, *P* < 0.01] (Fig 2E) and females [t = 4.47, *P* < 0.001] (Fig 2F), indicating that consuming a HFD produced a significant increase in leptin levels in WT male and female mice. Additionally, leptin level was higher in WT HFD than KO HFD males [t = 2.59, *P* < 0.05] (Fig 2E), in accordance with greater cumulative HFD intake in WT males than KO males. Circulating leptin levels were not significantly different between KO LFD and KO HFD males [t = 1.30, *P* > 0.05] (Fig 2E) or females [t = 1.65, *P* > 0.05] (Fig 2F). Thus HFD-induced leptin increase was attenuated in KO mice. The ANOVAs suggested trend but yielded no significant interaction between diets and genotypes for males [F(1, 31) = 3.30, *P* = 0.08] and females [F(1, 35) = 3.76, *P* = 0.06].

### CORT levels during acute stress or no stress control condition

Circulating CORT levels increased in all no stress control (NS) groups and acute restraint stress (S) groups regardless of sex, genotype, or diet, likely due to repeated multiple, serial blood sampling (Fig 3), as has been demonstrated in rodents [32]. LFD-fed males subjected to restraint stress (LFD/S) had a greater increase in CORT levels compared to LFD-fed no stress (LFD/NS) males (Fig 3A). Specifically, CORT levels of WT LFD/S males were significantly higher than WT LFD/NS males towards the end of the restraint stress (30 min) [t = 3.35, *P* < 0.01] and remained high 30 min after WT LFD/S mice were returned to their home cages (60 min) [t = 3.42, *P* < 0.01]; CORT levels of KO LFD/S males were significantly higher than KO LFD/NS males at 15 min [t = 3.16, *P* < 0.01] and 30 min [t = 4.70, *P* < 0.001] during the stress (Fig 3A). In contrast, CORT levels were similar between HFD/NS and HFD/S groups of WT and KO males (Fig 3B), suggesting that consumption of a HFD diminished stress response regardless of genotype in males. Indeed, when WT LFD and HFD groups were compared, CORT levels were higher in male LFD/S than LFD/NS at 30 min [t = 4.40, *P* < 0.001] and 60 min [t = 4.50, *P* < 0.001], but were not significantly different between HFD/NS and HFD/S at any time point (Fig 3C). For male KO groups, CORT levels were higher in male LFD/S than LFD/NS at 30 min [t = 3.84, *P* <0.001], but were similar between HFD/NS and HFD/S throughout the test (Fig 3D). Different from males, HFD consumption did not suppress the HPA stress response in females. CORT levels of LFD/S and HFD/S females were higher than their same genotype and same diet NS counterparts. Specifically, CORT levels were higher in both WT LFD/S [t = 3.02, *P* < 0.05] and KO LFD/S [t = 5.56, *P* < 0.001] females compared to their same genotype LFD/NS groups at 30 min (Fig 3E). CORT levels were also higher in WT HFD/S females than WT HFD/NS females at 30 min [t = 3.56, *P* < 0.01] and 60 min [t = 2.66, *P* < 0.05], and higher in KO HFD/S females than KO HFD/NS females at 15 min [t = 4.54, *P* < 0.001], 30 min [t = 5.48, *P* < 0.001], and 60 min [t = 5.51, *P* < 0.001] (Fig 3F). When WT LFD and HFD groups were compared, CORT levels were higher in female LFD/S than LFD/NS at 30 min [t = 2.94, *P* <0.05], and higher in female HFD/S than HFD/NS at 30 min [t = 3.61, *P* <0.01] and 60 min [t = 2.70, *P* <0.05]. (Fig 3G). For female KO groups, CORT levels were higher in female LFD/S than LFD/NS at 30 min [t = 5.51, *P* <0.001], and higher in female HFD/S than HFD/NS at 15 min [t = 4.69, *P* <0.001], 30 min [t = 5.65, *P* <0.001], and 60 min [t = 5.68, *P* <0.001] (Fig 3H).

Additionally, CORT levels were higher in KO males than their WT counterparts. CORT levels were higher in KO LFD/NS males than WT LFD/NS males at 60 min [t = 2.88, *P* < 0.05] (Fig 3A), and higher in KO HFD males than WT HFD males within the same stress condition at 30 min [NS: t = 3.46, *P* < 0.001; S: t = 4.11, *P* < 0.001] and 60 min [NS: t = 5.56, *P* < 0.001; S: t = 5.41, *P* < 0.001] (Fig 3B). Similarly, some female KO groups had higher CORT levels than their WT counterparts with the same diet and stress condition. Specifically, CORT levels were higher in KO LFD/S females than WT LFD/S females at 30 min [t = 2.71, *P* < 0.05] (Fig 3E), and higher in KO HFD/S females than WT HFD/S females at 15 min [t = 3.21, *P* < 0.01] and 30 min [t = 4.88, *P* < 0.001] (Fig 3F). Thus, the lack of PRL led to higher levels of CORT under both non-stressed and stressed conditions, most evidently in HFD-fed males and females.

### Hypothalamic CRH levels

**Fig 4:**
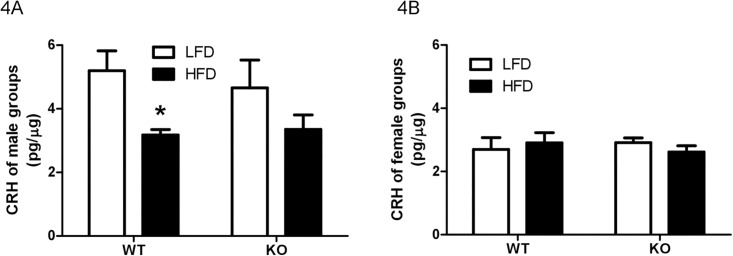
Hypothalamic corticotrophin releasing hormone levels. Hypothalamic corticotrophin releasing hormone (CRH) levels of male (A) and female (B) wildtype (WT) or prolactin knockout (KO) mice fed with a low-fat diet (LFD) or a high-fat diet (HFD). * Different between diets with the same genotype.

The only significant effect of diet or genotype on hypothalamic CRH levels was that HFD feeding produced a decrease in hypothalamic CRH content in WT males [t = 2.56, *P* < 0.05] (Fig 4A). Although, a similar pattern occurred in KO male mice, there was no significant difference [t = 1.66, *P* = 0.09] (Fig 4A). There was a main effect of diet [F(1,40) = 8.89, *P* = 0.0049] but not genotype [F(1,40) = 0.11, *P* = 0.75], and there was no interaction between diet and genotype for male CRH level [F(1,40) = 0.41, *P* = 0.53]. Hypothalamic CRH content was similar among all groups of females (Fig 4B), and there was no main effect of diet [F(1,29) = 0.03, *P* = 0.87] or genotype [F(1,29) = 0.02, *P* = 0.89], and no interaction between them [F(1,29) = 0.94, *P* = 0.34].

### PRLR expression in the choroid plexus

**Fig 5:**
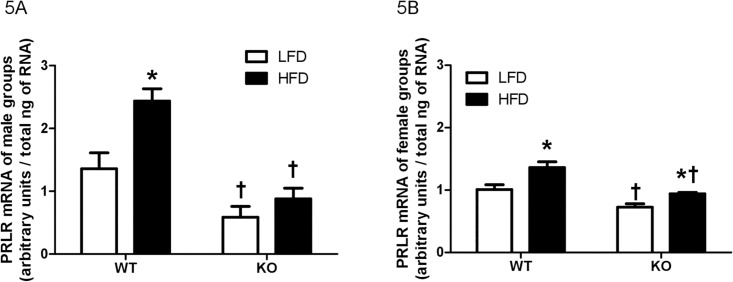
Prolactin receptor mRNA levels in the choroid plexus. Prolactin receptor (PRLR) mRNA levels in the choroid plexus of male (A) and female (B) wildtype (WT) or prolactin knockout (KO) mice fed with a low-fat diet (LFD) or a high-fat diet (HFD). * Different between diets with the same genotype. † Different between genotypes with the same diet.

Regardless of sex and diet, when compared with the same-sex WT counterparts, PRLR mRNA levels were significantly lower in KO males [LFD: t = 2.80, *P* < 0.05; HFD: t = 5.40, *P* < 0.001] (Fig 5A) and KO females [LFD: t = 3.16, *P* < 0.05; HFD: t = 4.66, *P* < 0.001] (Fig 5B). Consumption of a HFD produced a significant increase in PRLR mRNA levels in WT males [t = 3.63, *P* < 0.01] but not in KO males [t = 1.10, *P* > 0.05] (Fig 5A), whereas it increased PRLR expression in both genotypes [WT: t = 3.71, *P* < 0.01; KO: t = 2.55, *P* < 0.05] in female groups (Fig 5B).

## Discussion

Although best characterized for its roles in the initiation and maintenance of lactation in mammals, PRL also attenuates the neuroendocrine response to stress [6,10,11] and has metabolic actions related to the regulation of body weight [1,2]. Chronic HFD feeding disrupts energy homeostasis and the HPA axis response to stress. The goal of this study was to determine the effects of PRL on the HPA axis response to stress during HFD feeding comparing LFD- and HFD-fed male and female WT and PRL KO mice. A wide array of hormones and tissues of PRL KO mice have been examined [26]. PRL deficiency leads to pituitary hyperplasia and undetectable PRL bioactivity in pituitaries [26]. Other pituitary hormones and tissues are normal in PRL KO mice [26]. Baseline levels of pituitary ACTH protein (data not shown), circulating corticosterone (Fig 3), and hypothalamic corticotrophin releasing hormone (Fig 4) were similar between WT and KO groups of the same sex and fed the same diet. Thus, there was no underlying abnormality due to PRL deficiency at baseline that would evoke differential HPA axis response. CORT levels during acute stress tests were higher in KO mice than their WT counterparts, suggesting greater HPA activation in mice lacking PRL, thus confirming PRL’s role in dampening the stress-induced HPA response. When PRL is absent, mice were more susceptible to stress-induced HPA activation. Basal PRLR mRNA levels in the choroid plexus were higher in HFD than LFD same-sex groups for both males and females, suggesting potential activation of central PRL action by HFD feeding. This result is consistent with the hypothesis that PRL acts as a regulator of HPA axis activity through central mechanisms [11]. For example, infusion of ovine PRL into the lateral cerebral ventricle produces anxiolytic effects, whereas down-regulation of PRLR following treatment with antisense oligonucleotide produces elevated anxiety- like behaviors [6,11].

The results from this study suggested that sex differences in body weight regulation and stress-induced HPA axis activation was mainly influenced by HFD feeding, but not by genotype. First, consuming a HFD significantly increased body weight and adiposity in males but not females, regardless of genotypes. Consistent with the literature, male rodents typically gain more weight and fat mass than females when fed with a HFD, possibly due to estrogen’s roles in decreasing lipid storage [33]. Second, although basal CORT levels were unaltered by HFD consumption in males or females, which is consistent with a previous study in which morning CORT levels were not changed after 3-months of HFD feeding in male mice [18], HFD feeding attenuated the CORT response to an acute stress in males but persisted in females, regardless of genotype. Plasma ACTH levels were measured and its increase during restraint stress was similar between genotypes for both sexes (data not shown). Circulating plasma ACTH levels may not reflect ACTH pulsatile secretion from the pituitary, however. Hypothalamic CRH content was quantified in the current study, because PRL may directly modulate CRH expression as PRLR are expressed in parvocellular neurons in the PVN [25]. Hypothalamic CRH content was lower in HFD-fed compared to chow-fed male mice, consistent with a previous study in which consumption of a HFD decreased CRH mRNA level in the PVN of male mice with no change in CORT levels [18]. Thus, the reduced HPA axis activation during stress in male HFD groups, indicated by attenuated CORT response, may not be due to lack of ACTH stimulation to the adrenal gland, but could be centrally driven. In the current study, HFD feeding decreased hypothalamic CRH content in males, whereas HFD-fed females maintained similar levels of CRH, possibly due to modulatory effects of estrogens on CRH transcription via estrogen receptor β in the hypothalamus [34] that conserves hypothalamic CRH at a constant level.

Sex differences exist in stress-induced HPA activation in rodents, with females having greater increases in ACTH and CORT levels than males [35]. Sex steroids possibly regulate the activity of the HPA axis. For example, enhanced HPA response to stress is greater during the proestrous phase of the ovarian cycle when estradiol levels are the highest [36,37]. Estradiol treatment increases glucocorticoid negative feedback and corrects disturbances in HPA axis activity in aging male rats [38]. Ovariectomy in females decreases [39,40], whereas castration in males increases [40], the secretion of CORT and ACTH to physical and psychological stressors; this is reversed by replacement of gonadal steroids after gonadectomy [40].

Leptin is a hormone synthesized in adipose tissues in proportion to adiposity and involved in the regulation of energy balance [41]. Leptin reduces the sensitivity of the adrenal cortex cells to ACTH and reduces CORT levels *in vitro* [42,43]. Also, increased leptin in response to HFD feeding reduced HPA axis activity in male rats *in vivo* [13], supporting the idea that the high leptin level in HFD-fed males eliminates the CORT response to stress. HFD feeding abolished the stress-induced CORT response and reduced hypothalamic CRH levels in males but not females, a sex difference possibly due to greater increases in leptin levels by HFD feeding in males than females. Consistent with a previous study [28], leptin levels were significantly higher in HFD WT mice. Interestingly, the increase circulating leptin level induced by HFD feeding was attenuated in PRL KO males and females, suggesting a stimulatory effect of PRL on leptin release in HFD-fed males and females.

It remains unclear how PRL regulates leptin levels independent of the adiposity level, however. Variable effects of PRL on leptin levels have been reported. For example, female mice that overexpress PRL [24] and male mice with PRL deficiency [28] have elevated leptin levels whereas female mice with PRL deficiency have similar leptin levels compared with their WT littermates [28] *in vivo*. The literature on direct effects of PRL on leptin production by adipocytes *in vitro* is also conflicting. PRL alone has no effect on leptin production in adipocytes of female mice [24]; whereas PRL dose-dependently inhibits leptin release from mature adipocytes of male rats [44]. These discrepancies may be due to differences in development of genetic mouse models, doses and time course of PRL, or adipocyte depot-specific leptin release. In the current study, no differences in leptin levels were observed between female genotypes in either LFD or HFD groups, or between male genotypes in LFD group. As expected, leptin was significantly higher in male and female WT mice on the HFD. Our data do not support the report on elevated leptin levels in LFD male KO mice, but was consistent with the similar leptin levels in WT and KO females from the same study [28]. Additionally, our finding showing suppressed or not significantly different leptin levels in PRL KO mice agrees with findings from studies using PRLR-deficient mouse models, showing PRLR-deficient mice with either lower [29] or similar [30] leptin levels comparing with their WT littermates. In general, the current study indicates that PRL deficiency has minor effects on leptin levels, which does not reflect global changes in weight gain, body composition, or adiposity in mice of either sex (Figs 1 and 2).

Similar to circulating PRL that enters the brain via PRLR in the choroid plexus [7–9], leptin enters the brain through specific receptors in the choroid plexus and median eminence, and stimulates anorexic pathways and inhibits orexigenic pathways within the CNS [45]. Also similar to PRL, peripheral or central administration of leptin attenuates the stress-induced secretion of hypothalamic CRH and plasma ACTH and CORT, and suppresses the stress-induced activation of the HPA axis in mice and rats [46–48]. Activity of the HPA axis is elevated in *ob/ob* mice that lack leptin [49] and peripheral leptin administration decreases circulating level of CORT in *ob/ob* mice [50]. These previous studies collectively suggest that HFD feeding increases leptin levels and leptin suppresses stress-induced HPA activation in a similar way as PRL.

PRL KO mice express functional PRLR [26]. PRLR mRNA levels in the choroid plexus were quantified because PRL regulates HPA axis activation by entering the brain through PRLR in the choroid plexus and acting on PRLR in the PVN during stress [8,11], and stress increases PRLR mRNA levels in the choroid plexus [12,51]. PRLR mRNA levels in the choroid plexus were lower in KO mice that lack PRL than WT mice, supporting previous findings that PRLR expression is regulated by circulating levels of PRL [7–9]. Sex difference in PRLR expression in the choroid plexus has been reported in rats, although PRLR mRNA levels were similar between LFD-fed male and female mice in the current study. The levels of PRLR mRNA in the choroid plexus are higher in female than male rats, and are higher during proestrus when endogenous estradiol reaches its peak than during other phases of the ovarian cycle in female rats [52]. Ovariectomized female rats treated with a pharmacological dose of estradiol (35 μg), but not with a physiological dose of estradiol (3.5 μg), have higher PRLR mRNA levels in the choroid plexus than vehicle-treated ovariectomized rats [53]. Thus whether or not sex difference and/or cyclic change of PRLR expression in the choroid plexus is caused by estradiol upregulation awaits further investigation. Consuming a HFD significantly increased PRLR mRNA in WT and KO females and in WT males in the current study, indicating that factors other than PRL, including diet, regulate PRLR expression. To our knowledge, this is the first report showing increased PRLR mRNA levels following consumption of a HFD. Increase in PRLR mRNA suggests that enhanced PRL signaling could be used to dampen HPA axis activation when consuming a HFD.

In conclusion, this study investigated the role PRL plays in regulating the HPA axis under consumption of HFD in response to an acute stress and potential sex differences using male and female PRL WT and KO mice. Sex differences existed in response to HFD consumption and effects of HFD on activation of the HPA axis by stress. First, there was sex differences in body weight gain and fat accumulation, whereas PRL did not play an important role in mediating these effects. PRL, however, acted as a regulator of the HPA axis by dampening stress effect induced by multiple blood sampling at least in males, as CORT levels were higher in KO than WT males. Second, there was sex difference in the HPA response to stress under the different dietary conditions. Consuming a HFD diminished the stress response in males regardless of genotype, whereas it did not affect stress response in females. Consuming a HFD was a chronic stressor because PRLR mRNA was increased in both WT and KO females and in WT males, suggesting that factors other than PRL regulate PRLR mRNA levels. These comprehensive studies indicate that although PRL deficiency has minor effects on caloric intake, circulating leptin levels, and PRLR expression in the choroid plexus, it does not result in significant changes in weight gain, adiposity, or stress response in mice of either sex.
